# Co-delivery of gambogic acid and TRAIL plasmid by hyaluronic acid grafted PEI-PLGA nanoparticles for the treatment of triple negative breast cancer

**DOI:** 10.1080/10717544.2017.1406558

**Published:** 2017-11-24

**Authors:** Shengpeng Wang, Min Shao, Zhangfeng Zhong, Anqi Wang, Jiliang Cao, Yucong Lu, Yitao Wang, Jinming Zhang

**Affiliations:** a State Key Laboratory of Quality Research in Chinese Medicine, Institute of Chinese Medical Sciences, University of Macau, Macau, China;; b Department of Bioengineering, Zunyi Medical University Zhuhai Campus, Zhuhai, Guangdong, China;; c College of Pharmacy, Chengdu University of Traditional Chinese Medicine, Chengdu, Sichuan, China

**Keywords:** Nanoparticles, TRAIL, gambogic acid, triple negative breast cancer, co-delivery

## Abstract

Tumor necrosis factor-related apoptosis-inducing ligand (TRAIL)-based combination therapy and gene therapy are new strategies to potentially overcome the limitations of TRAIL, however, the lack of efficient and low toxic vectors remains the major obstacle. In this study, we developed a hyaluronic acid (HA)-decorated polyethylenimine-poly(d,l-lactide-co-glycolide) (PEI-PLGA) nanoparticle (NP) system for targeted co-delivery of TRAIL plasmid (pTRAIL) and gambogic acid (GA) in triple-negative breast cancer (TNBC) therapy. GA was encapsulated into the core of the PEI-PLGA NPs while pTRAIL was adsorbed onto the positive NP surface via charge adsorption. The coating of HA on PEI-PLGA NPs functions as a targeting ligand by binding to CD44 receptor of TNBC cells and a shell to neutralize the excess positive charge of inner NPs. The resultant pTRAIL and GA co-loaded HA-coated PEI-PLGA NPs exhibited spherical shape (121.5 nm) and could promote the internalization of loaded cargoes into TNBC cells through the CD44-dependent endocytic pathway. The dual drug-loaded NPs significantly augmented apoptotic cell death *in vitro* and inhibited TNBC tumor growth *in vivo*. This multifunctional NP system efficiently co-delivered GA and pTRAIL, thus representing a promising strategy to treat TNBC and bringing forth a platform strategy for co-delivery of therapeutic DNA and chemotherapeutic agents in combinatorial TNBC therapy.

## Introduction

Tumor necrosis factor-related apoptosis-inducing ligand (TRAIL, also known as APO2L or TNFSF10), a member of the tumor necrosis factor (TNF) superfamily, was identified in the mid-1990s based on the homologic sequence to TNF and CD95L (Wiley et al., 1995). Unlike TNF, TRAIL induces apoptosis in various human cancer cells but does not kill normal cells. TRAIL mainly interacts with two distinct receptors, namely, TRAILR1 (also known as death receptor 4, DR4) and TRAILR2 (also known as DR5), and recruits Fas-associated death domain protein (FADD) and procaspase-8/10 to form the death-inducing signaling complex (DISC) (Kischkel et al., 2000; Kischkel et al., 2001). The formation of DISC activates caspase 8 and the cleaved caspase 8 directly induce cell apoptosis via activation of the downstream substrates of the apoptotic pathway. The discovery of TRAIL rapidly excited its entry into clinical trials. Data from clinical trials of recombinant human TRAIL showed well tolerability, however, TRAIL failed to produce expected therapeutic activity (Farooqi et al., 2016). Meanwhile, many cancer cells are either inherent resistant to or can develop resistance to TRAIL-induced cell death (Lemke et al., 2014). In addition, the heterogeneous tumor microenvironment and the rapid *in vivo* clearance also pose various barriers to delivering sufficient proteins to the targeted tumor cells (MacFarlane, 2003; Prasad et al., 2014).

The resistance of human cancers to TRAIL-mediated apoptosis has prompted the attempts to develop TRAIL-based combination therapies with superior efficacy. Many chemotherapeutic agents and natural products have showed the potential to sensitize tumor cells to overcome TRAIL resistance (Dorsey et al., 2009; Wang et al., 2010; Allensworth et al., 2012). Up-regulation of the expression of DRs, activation of DISC-mediated signaling, and suppression of inhibitor proteins of the caspase cascade have been proposed as the mechanistic basis of TRAIL sensitization (Tsai et al., 2006). Meanwhile, in contrast to TRAIL protein therapy, TRAIL-based gene therapy can potentially overcome the limitations of TRAIL by delivering TRAIL encoding DNA specifically to tumor cells and promoting the expression and secretion this therapeutic protein into the tumor microenvironment (Kagawa et al., 2001; Luo et al., 2016). Delivery of drug-nucleic acid combinations to the same tumor cell populations by one single carrier has been validated as a promising strategy for enhancement of cancer treatment (Li et al., 2013). Co-delivery of these drug-nucleic acid combinations can tailor multiple disease pathways, optimize their dramatically different pharmacokinetic profiles and biodistribution behaviors, resulting in a more efficient synergistic antitumor activity (Teo et al., 2016). Emerging new evidence suggests the successful delivery of TRAIL-based combination therapies using a single carrier to achieve a more efficient combinatorial antitumor effect (Wu et al., 2017).

**Figure 1.**
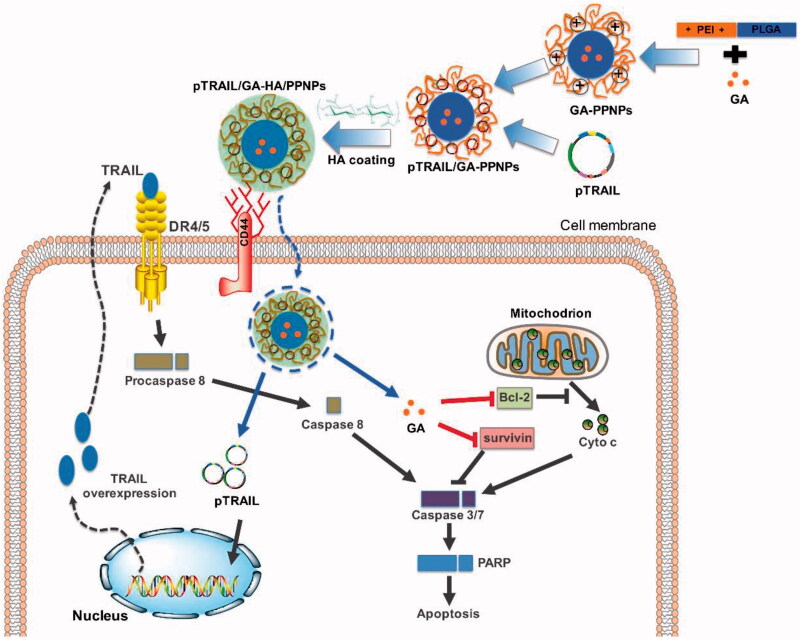
Schematic fabrication of HA grafted PEI-PLGA NPs for plasmid co-delivery of GA and TRAIL.

Triple-negative breast cancer (TNBC) that do not express estrogen receptor (ER), progesterone receptor (PR) and human epidermal growth factor receptor 2 (HER2) represent an aggressive subgroup of breast cancer (Brewster et al., 2014) with poor prognosis and limited treatment options in the clinic (Foulkes et al., 2010). In a previous study, we described a hyaluronic acid (HA)-decorated polyethylenimine-poly(d,l-lactide-co-glycolide) (PEI-PLGA) NP system for targeted co-delivery of chemotherapeutic agent doxorubicin (DOX) and tumor suppressive microRNA miR-542-3p in TNBC therapy (Wang et al., 2016), are deemed the subgroup of breast cancer patients with the worst outcome (Foulkes et al., 2010; Turner & Reis-Filho, 2013). In this system, PLGA and PEI, two widely studied polymers were utilized for drug and gene delivery, respectively (Lei et al., 2013). However, the excess positive charge of PEI-PLGA NPs will result in side effects because of the interaction with hemoglobin in blood circulation and normal tissues. The natural polysaccharide HA with tumor-specific targeting properties due to its high affinity toward CD44 receptor, a cell adhesion membrane glycoprotein overexpressed in cancer cells (Aruffo et al., 1990) was applied to achieve negative surface charge and TNBC active targeting effect. We and other groups have showed the potential of gambogic acid, a natural compound acts on multiple targets, in potentiating the effect of various first-line chemotherapeutic agents for the treatment of breast cancer (Wang et al., 2014; Wang et al., 2015). In this study, the nonviral vector of HA-coated PEI-PLGA NPs was further developed for simultaneously delivering GA and a plasmid encoding TRAIL (pTRAIL) (graphic illustrated in Figure 1). The therapeutic effects and mechanisms of the nanoparticulate system in *in vitro* and *in vivo* models of TNBC were further investigated.

## Materials and methods

### Chemicals and reagents

GA with purity over than 95% was purchased from Chengdu Must Bio-Technology Co. Ltd (Sichuan, China). A 5.6-kbp TRAIL-enhanced green fluorescent protein (GFP) expression plasmid (pEGFP-TRAIL, Addgene, MA) was amplified in *Escherichia coli* strain DH5α and purified using the HiSpeed Plasmid Maxi Kit (Qiagen, Valencia, CA). HA with an average Mw of 170 kDa and PLGA of 5000 average Mw were supplied by Lifecore Biomedical (San Francisco, CA) and Jinan Daigang Biomaterial Co., Ltd. (Shandong, China), respectively. Linear PEI (average Mw 5000), 3-(4,5-dimethylthiazol-2-yl)-2,5-diphenyltetrazolium bromide (MTT) and paraformaldehyde (PFA) were supplied by Sigma-Aldrich (St. Louis, MO). Dulbecco’s Modified Eagle Medium (DMEM), fetal bovine serum (FBS), penicillin-streptomycin and 0.25% (w/v) trypsin/1 mM EDTA were purchased from Life Technologies (Grand Island, NE). The primary antibodies against cleaved PARP, cleaved caspase 3, caspase 8, survivin, Bcl-2 and GAPDH were purchased from Cell Signalling Technology (Danvers, MA).

### Cell lines and animals

Human breast cancer MCF-7 and MDA-MB-231 cell lines, and mouse mammary breast tumor 4T1 cell line were obtained from the American Type Culture Collection (ATCC, Manassas, VA). Cells were cultured in DMEM medium supplemented with 10% FBS and penicillin/streptomycin (100 U/mL; 100 μg/mL) in a 37 °C, 5% CO_2_ incubator. Female BALB/c mice (18 ± 2 g) were obtained from Guangdong Medical Laboratory Animal Center (China). Animal experiments were performed according to the protocol approved by Institutional Animal Care and Use Committee, Zunyi Medical University.

### Preparation of NPs

The PEI-PLGA copolymer was synthesized and characterized as our previous study reported (Wang et al., 2016). PEI-PLGA NPs (PPNPs) with or without GA loading were prepared by dialysis method. Briefly, 10 mg of PEI-PLGA copolymer with or without 1 mg of GA was dissolved in 1 mL DMSO. After ultrasonic mixing for 1 min, this mixture was introduced into a dialysis tube with MWCO of 3500 Da and dialyzed against PBS buffer for 8 h. To further load pTRAIL, various amounts of pTRAIL were added into the PPNP suspension to yield different N/P ratios. The N/P ratio, which determines the ratio of nitrogens (N) of PEI polymer to phosphates (P) of pTRAIL, was expressed as the PPNPs/DNA ratio. These complexes were mixed at 25 °C for 0.5 h. Gel electrophoresis was then employed to optimize the suitable feeding amount of pTRAIL in the NP suspension. After filtration through a 0.45 μm filter, the obtained GA/pTRAIL-loaded PPNPs were stored at 4 °C for further studies. Finally, according to our previous study, the appropriate amount of HA was dropwise added into NP suspension with the ratio of HA: NPs at 1.5: 10 (wt/wt) to neutralize the excess positive charge of PPNPs (Wang et al., 2016). Eventually, GA/pTRAIL-HA/PPNPs were obtained by further filtration through a 0.45 μm filter.

### Characterization of NPs

The average size, size distribution and zeta potential of NPs with or without HA coating were measured by dynamic light scattering (DLS) using a Nano-ZSP ZetaSizer (Malvern Instruments, Malvern, UK). Each batch was analyzed in triplicate. Morphological examination of GA/TRAIL-HA/PPNPs was performed by transmission electron microscopy (TEM) (Tecnai G20, FEI, Hillsboro, OR) at an accelerating voltage of 200 kV.

To determine the GA-loading efficiency, the drug-loaded HA/PPNP formulation was disrupted by acetonitrile to release the encapsulated drug. The amount of GA entrapped in the NPs was estimated by Waters e2695 high performance liquid chromatography (HPLC) equipped with a reverse phase C18 column (150 × 4.6 mm, 5 μm) at a flow rate of 1 mL/min. The mobile phase for GA detection was acetonitrile: water containing 0.25% acetic acid (26: 74, v/v). GA encapsulation efficiencies (EE) and drug loading (DL) were calculated using the following equations:

$$EE\;of\;GA\;=\frac{weight\;of\;GA\;in\;the\;NPs}{weight\;of\;feeding\;GA}\times \;100\%$$

$$DL\;of\;GA=\frac{weight\;of\;GA\;in\;the\;NPs}{weight\;of\;the\;GA-loaded\;NPs}\times \;100\%$$

The EE of pTRAIL in NPs was measured similarly. The non-entrapped pTRAIL in the aqueous phase was separated by the ultrafiltration method (30 kD, 30 min). For analysis of pTRAIL, a cell impermeant nucleic acid dye, YOYO1, was applied to label pTRAIL. The concentration of YOYO1-pTRAIL (*λ*_ex_ 494 nm and *λ*_em_ 509 nm) was measured using a Lumina Fluorescence Spectrometer (Thermo Scientific).

### Gel electrophoresis

Gel electrophoresis was carried out using a 1.5% agarose gel containing 0.5 μg/mL GelRED at 80 V for 10 min in Tris-acetate-EDTA (TAE) buffer. The gel was subsequently photographed using the ChemiDocTM Imaging System (Bio-Rad, Hercules, CA).

### Cellular uptake study

The intracellular drug levels were evaluated by flow cytometry and fluorescence microscopy. Briefly, cells were properly treated and the medium was then removed and cells were carefully washed three times with PBS. For observation by fluorescence microscopy, cells were fixed with 4% PFA, washed again with PBS, stained with Hoechst 33342 (1 μg/mL), and imaged using Incell Analyzer 2000 (GE Healthcare Life Sciences, Pittsburgh, PA, USA). Each condition was performed in triplicate. For flow cytometric analysis, cells were collected and washed thrice with PBS, then analyzed using a flow cytometer (BD Biosciences). Generally, a total of 10,000 cells were collected, amplified, and scaled to generate single parameter histogram.

### Cell viability assay

Cell viability was assessed by MTT assay. Briefly, exponentially growing cancer cells were seeded in 96-well plates and incubated overnight. After appropriate treatment, the cells incubated with serum-free medium containing MTT (1 mg/mL) for another 4 h. The formazan crystal was dissolved using DMSO and the spectrophotometric absorbance at 570 nm was determined by a microplate reader (SpectraMax M5, Molecular Devices, USA). The results were expressed as the percentage of viable cells over untreated control cells.

### Determination of apoptosis

Apoptosis was evaluated using an Annexin V-FITC Apoptosis Detection Kit (BioVision, Palo Alto, CA) by flow cytometry. Briefly, after appropriate treatment, the cells were collected by EDTA-free trypsin, washed twice with PBS and then stained with 5 μL of Annexin V-FITC and 10 μL of PI for 20 min at room temperature in the dark prior to flow cytometric analysis.

### Caspase activity assay

Caspase 3/7 and caspase 8 activities were measured using the Caspase-Glo assay kit (Promega, Madison, WI). Cells were plated into 96-well white-walled plates and appropriately treated for 24 h. Subsequently, 100 μL of caspase assay reagent buffer was added to each well and incubated in the dark for 30 min. The bioluminescence was determined using a SpectraMax M5 microplate reader.

### Western blot analysis

After appropriate treatment, cells were washed three times with ice-cold PBS and extracted by RIPA lysis buffer containing 1% PMSF and 1% protease inhibitor cocktail for 30 min on ice, followed by centrifugation at 12,500 × *g* for 20 min at 4 °C. The protein concentration was determined using the BCA protein assay kit (Thermo Fisher Scientific, Waltham, MA, USA). Equivalent amounts of protein samples were separated by sodium dodecyl sulfate-polyacrylamide gel electrophoresis (SDS-PAGE) using 10% (w/v) gels, and then transferred to a polyvinylidene difluoride (PVDF) membrane (Bio-Rad, Hercules, CA). After blocking with 5% (w/v) nonfat milk for 1 h at room temperature, the blots were incubated with primary antibodies overnight at 4 °C. After washing for three times, the membranes were further incubated with corresponding secondary antibodies for 1 h at room temperature. Finally, specific protein bands were visualized using ECL Plus Western blotting detection reagents (GE Healthcare, Piscataway, NJ) and scanned by a ChemiDoc XRS Imaging system (Bio-Rad, Hercules, CA).

### In vivo *anti-tumor efficacy*

To observe therapeutic efficacy in animal model, tumor-bearing nude BALB/c mice (5 weeks old, 18–20 g, female) were prepared by inoculating 4T1 cells into the left flank of mice. When tumors grew to approximately 50–100 mm^3^ in volume, mice (*n* = 5) were divided into different groups and injected with various samples via tail vein. The therapeutic results of each group were evaluated by calculating the tumor volumes and the total weight of tumors. To evaluate toxicity caused by therapeutic agents, the body weight, consumption of water and food in each animal group were also measured during the study.

Tumor volume (V) and tumor suppression rate (TSR) were calculated based on the following equation:

$$V=a\times b^{2}/2$$

$$TSR={(V}_{c}-V_{x})/V_{c}\times 100\%$$

where a and b are the length and width axes of the tumors measured by caliper. V_c_ and V_x_ represents tumor volume of the control group and the treatment group, respectively.

For histological analysis, tumor tissue and organs were excised from mice and fixed with 4% PFA solution and embedded in paraffin after treatment. The sliced tumor tissue and organs were further subjected to hematoxylin and eosin (H&E) staining and terminal deoxynucleotidyl transferase-mediated dUTP-biotin nick end-labeling (TUNEL) staining.

### Statistical analysis

Statistical analysis was performed using the GraphPad Prism 6.0 statistical software (San Diego, CA). The results were expressed as the mean of arbitrary values ± standard error mean (SEM). Statistical significance was assessed by one-way ANOVA followed by Tukey’s multiple comparison, where a *p*-value less than .05 denoted statistical significance.

## Results and discussion

### Preparation and characterization of GA- andpTRAIL-loaded HA/PPNPs

**Figure 2.**
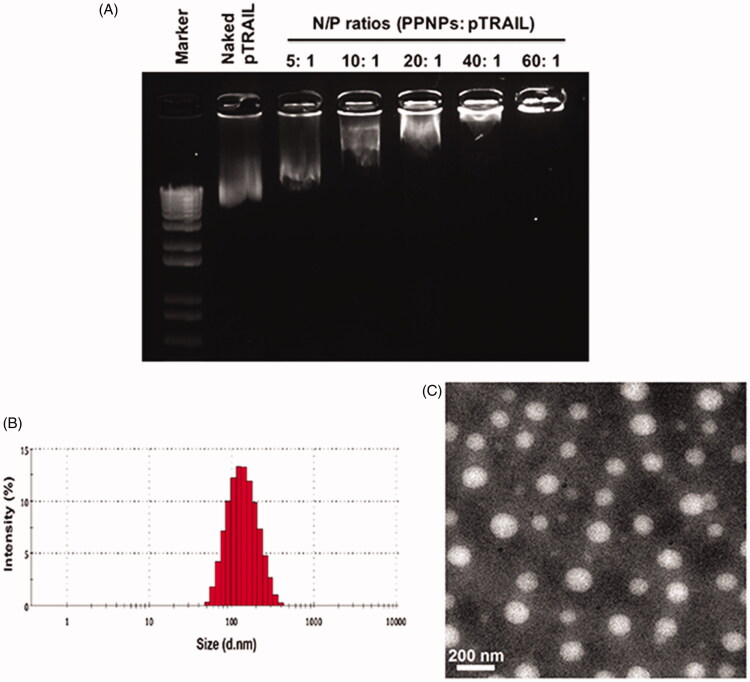
Preparation and characterization of GA and pTRAIL co-loaded HA/PPNPs. (A) Optimization of charge ratios (N/P) between pTRAIL and PPNPs was visualized by gel electrophoresis. Size distribution and particle morphology was measured by dynamic light scattering (B) and transmission electron microscopy (C).

Due to the amphiphilic structure of the hydrophilic linear PEI and the hydrophobic PLGA, the PEI-PLGA copolymer was further used to prepare micelle-like PPNPs. The PPNPs loaded GA into the core of NPs and carried pTRAIL through the binding with the cationic PEI moiety, successively. Firstly, GA was encapsulated into the PPNPs by a simple dialysis method. The ability of NPs to complex pTRAIL was then evaluated using gel retardation assay. Polyplex formation between PPNPs and pTRAIL was investigated at various N/P ratios ranging from 5 to 60. As shown in Figure 2(A), naked pTRAIL freely migrated on agarose gel, complete retardation of the pTRAIL was observed at an N/P ratio of 60.

However, although positively charged NPs can be readily internalized into cancer cells through nonspecific electrostatic interaction between the NPs and the negatively charged cell membranes, there is a major concern of potential side effects (Cao et al., 2011). We thus coated negative charged HA onto the surface of the PPNPs to neutralize the positive surface charge. Finally, with high encapsulation efficiencies (EE) and drug loading (DL) of GA (83.3 ± 3.2% and 8.5 ± 0.4%, respectively) and pTRAIL (EE at 77.5 ± 2.4%), the GA and pTRAIL co-loaded HA/PPNPs (GA/pTRAIL-HA/PPNPs) showed an average size of 121.5 nm, which was small enough to selectively accumulate in tumor tissue due to the presence of endothelial dysfunction and blood vessel fenestrations, namely, the enhanced permeability and retention (EPR) effect (Blanco et al., 2015). The surface charge of the GA/pTRAIL-HA/PPNPs was −0.6 mV. The images of size distribution and zeta potential determined by DLS were shown in Figure 2 and Table 1.

**Table 1.** Characterization of GA and pTRAIL loaded HA/PPNPs (*n* = 3).

Preparations	Size (nm)	PDI	Zeta potential (mV)	EE (%)	DL (w/w, %)
PPNPs	107.2 ± 2.0	0.12 ± 0.03	50.3 ± 5.2	–	–
HA/PPNPs	119.4 ± 5.8	0.16 ± 0.01	−7.3 ± 5.7	–	–
GA/pTRAIL-H	121.5 ± 5.9	0.16 ± 0.01	−0.6 ± 0.43	83.3 ± 3.2^a^	8.5 ± 0.4^a^
A/PPNPs				77.5 ± 2.4^b^	–

a EE or DL of GA.

b EE of pTRAIL.

### HA/PPNP-mediated targeted co-delivery of GA and pTRAIL

**Figure 3.**
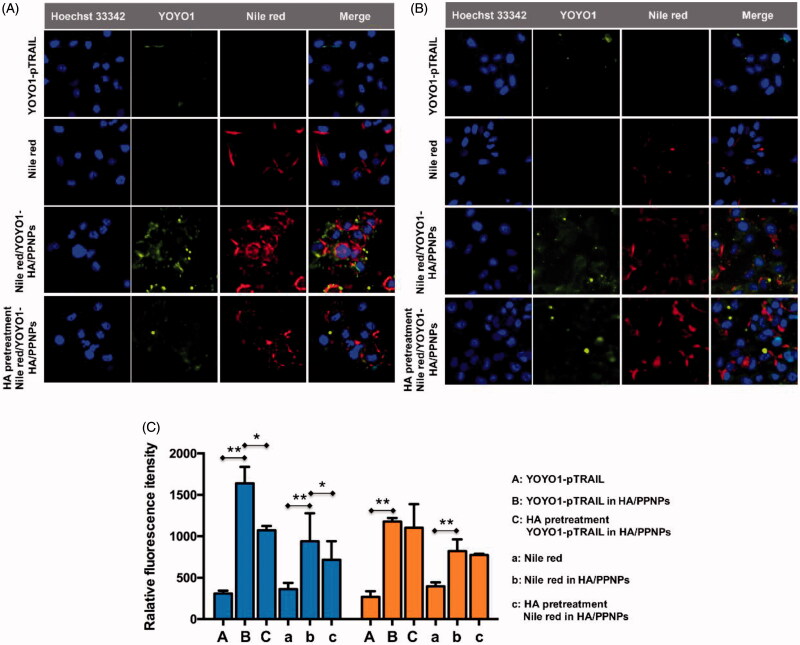
Intracellular uptake of HA/PPNPsin MDA-MB-231 cells. MDA-MB-231 (A) and MCF-7 (B) cells were incubated with nile red, naked YOYO1-pTRAIL, or nile red-and YOYO1-pTRAIL co-loaded HA/PPNPs with or without the pretreatment of 1 mg/ml of HA for 4 h, and the intracellular accumulation of nile red and YOYO1-pTRAIL was observed by the Incell Analyzer 2000 (GE Healthcare). (C) MDA-MB-231and MCF-7cellswere treated as described above and the intracellular fluorescence intensity was measured by flow cytometry. Data were expressed as the mean ± SEM of three independent experiments. *p < .05 and **p < .01.

As the major receptor of HA, CD44 receptors are responsible for binding to HA and resulting in active internalization of the loaded cargoes (Gotte & Yip, 2006). Metastatic breast carcinoma cell lines such as MDA-MB-231 and MDA-MB-435 are reported to upregulate the expression of CD44 compared with the nonmetastatic MCF-7 cell line (Vlodavsky et al., 1999; Surace et al., 2009). Our previous flow cytometry study showed a 20.5-fold higher CD44 expression in MDA-MB-231 cells compared to MCF-7 cells (Wang et al., 2016). Herein, MDA-MB-231 with and MCF-7 cells were employed to evaluate the targeting effect of HA-coated PPNPs in TNBC cells. To imitate HA/PPNP-mediated delivery of GA, lipophilic dye nile red was encapsulated into HA/PPNP while YOYO-1 was used to label pTRAI. Higher cellular uptake efficiency of nile red (100 ng/mL) and YOYO1-pTRAIL (1 μg/mL) was observed in drug loaded HA/PPNPs group compared with free nile red or naked YOYO1-pTRAIL in both MDA-MB-231 and MCF-7 cells (*p* < .01). To be specific, HA/PPNPs increased the fluorescence intensity of nile red and YOYO1-pTRAIL to 2.59- and 5.29-fold in MDA-MB-231 cells, respectively, comparing with free counterparts. Similar increase was also observed in MCF-7 cells. While the intensity of both nile red and YOYO1-pTRAIL in MDA-MB-231 cells was remarkably diminished after HA pretreatment (*p* < .05) (Figure 3(A,C)), resulting from the pre-saturation of CD44 receptors by free HA. In contrast, due to the low CD44 expression in MCF-7 cells, HA pretreatment did not block the increased cellular uptake of neither nile red or YOYO1-pTRAIL loaded in HA/PPNPsin MCF-7 cells (Figure 3(B,C)). Our results indicated that HA/PPNPs might promote the internalization of GA and pTRAIL into TNBC cells through the CD44-dependent endocytic pathway.

### Cytotoxicity of GA/pTRAIL-HA/PPNPs in breast cancer cells

**Figure 4.**
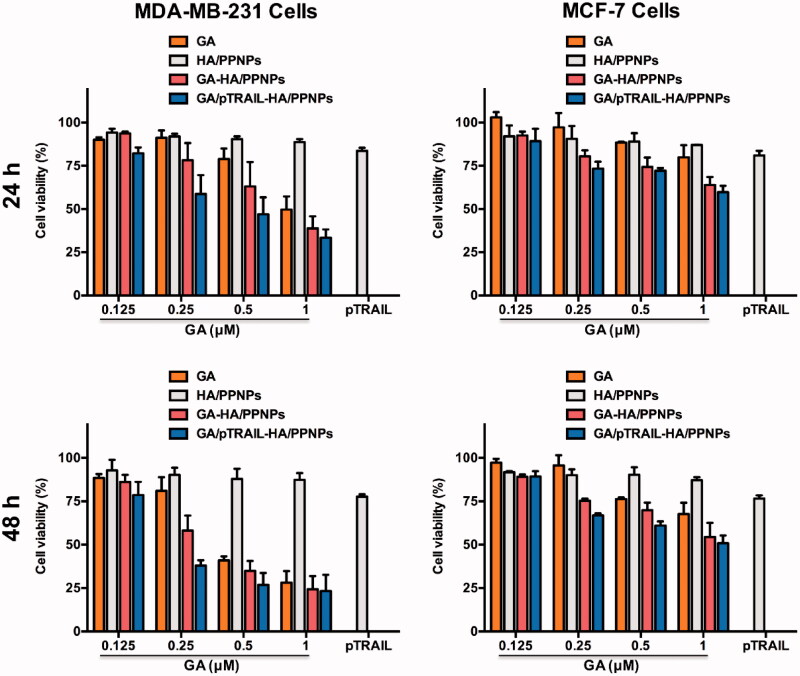
Cell viability of breast cancer cells after treatment with different formulations of GA and pTRAIL. MDA-MB-231 and MCF-7 cells were treated with different formulations of GA and pTRAIL for 24 h and 48 h. Cells viability was determined by MTT assay and compared to untreated cells. Data were expressed as the mean ± SEM of three independent experiments.

We further moved to verify whether HA/PPNPs co-loaded with GA and pTRAIL had synergistic effects against breast cancer cells. We compared the cytotoxicity of blank HA/PPNPs, free GA, GA-HA/PPNPs, pTRAIL-HA/PPNPs and GA/pTRAIL-HA/PPNPs against MCF-7 and MDA-MB-231 cells under equivalent concentrations of GA for 24 and 48 h, and the percentage of the viable cells was determined by MTT assay. As illustrated in Figure 4, HA/PPNPs did not significant decrease cell viability in either of the tested breast cancer cell lines. GA decreased the cell viability of both MDA-MB-231 and MCF-7 cells in a concentration- and time-dependent manner, and GA-loaded HA/PPNPs markedly increased the cytotoxicity of GA, which may be attributed to the increased cellular uptake. pTRAIL-loaded HA/PPNPs also induced a decrease in cell viability, while the highest cytotoxicity (27.0% survival) was observed in GA/pTRAIL-HA/PPNPs-treated cells, indicating a significant synergetic effectiveness for the combination of GA and pTRAIL. Meanwhile, GA/pTRAIL-HA/PPNPs induced more significant decrease of cell viability in MDA-MB-231 cells than MCF-7 cells at the same concentration. For instance, GA/pTRAIL-HA/PPNPs resulted in a more significant decrease of cell viability in MDA-MB-231 cells (62.0%) than that of MCF-7 cells (33.1%) at 0.25 μM, which might be attributed to the selective accumulation in CD-44 overexpressed MDA-MB-231 cells.

### Apoptosis analysis

**Figure 5.**
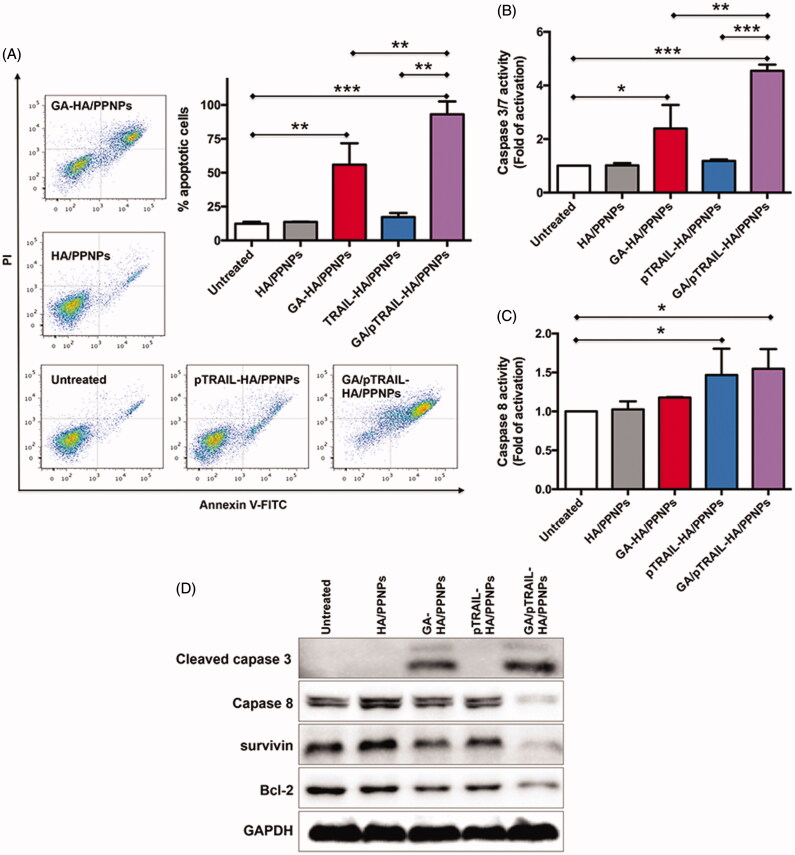
Cell apoptosis assay in TNBC cells. (A) Cell apoptosis was analyzed by flow cytometry using Annexin V-FITC/PI double staining. Involvement of caspase3/7 (B) and caspase 8 (C) activation. (D) Western blot analysis of apoptosis-related protein expression using indicated antibodies. Each value represents the mean ± SEM from triplicate determinations. *p < .05, **p < .01 and ***p < .001.

Cell apoptosis was subsequently monitored by flow cytometry. Cells maintained in standard culture medium were used as untreated controls. As shown in Figure 5(A), after treatment for 48 h, few apoptotic cells were detected in the untreated (12.4%) and HA/PPNPs-treated (13.6%) groups. A significant increase in cell apoptosis (55.92%) was found in cells treated with GA-HA/PPNPs. Furthermore, although pTRAIL-HA/PPNPs did not significantly promote cell apoptosis (17.3%), co-delivering GA and pTRAIL using HA/PPNPs induced almost all the cell apoptosis (93.1%). Data from caspase activity assay indicated that the caspase 3/7 activity of MDA-MB-231 cells was not significantly increased by blank HA/PPNPs, while increases of 2.39-, 1.18- and 4.55-fold were observed after the 24 h treatment with GA-HA/PPNPs, pTRAIL-HA/PPNPs and GA/pTRAIL-HA/PPNPs, respectively (Figure 5(B)). For caspase 8, only pTRAIL-HA/PPNPs and GA/pTRAIL-HA/PPNPs increased the activity of caspase 8 by 1.47- and 1.55-fold as compared to untreated control, meaning that the introduction of pTRAIL activated caspase 8 (Figure 5(C)). We further confirmed these results by observing the cleavage products of caspases. GA/pTRAIL-HA/PPNPs showed the strongest ability in promoting the cleavage of both caspase 3 and caspase 8 (Figure 5(D)). For anti-apoptotic proteins, treatment of GA/pTRAIL-HA/PPNPs led to the lowest expression level of survivin and Bcl-2.

### *In vivo* anti-tumor effect of GA/pTRAIL-HA/PPNPs

After observing the anticancer activities of GA/pTRAIL-HA/PPNPs in *in vitro* studies, we moved forward to evaluate their antitumor effect in an *in vivo* animal tumor model. The mouse 4T1 cell line was used to establish the TNBC tumor model in BALB/c mice. When subcutaneous 4T1 tumors grew to about 50 mm^3^ in volume, the tumor-bearing mice were divided into five groups (*n* = 5): (i) saline, (ii) empty HA/PPNPs, (iii) GA-HA/PPNPs (3 mg/kg of GA), (iv) pTRAIL-HA/PPNPs (20 μg of pTRAIL/mouse), (v) GA/pTRAIL-HA/PPNPs (3 mg/kg of GA and 20 μg of pTRAIL/mouse). Drugs were intravenously administrated via tail vein into 4T1-bearing mice on day 1, 3, 5, 7 and 9. The therapeutic efficacy was observed by measuring tumor volumes for 15 days.

**Figure 6.**
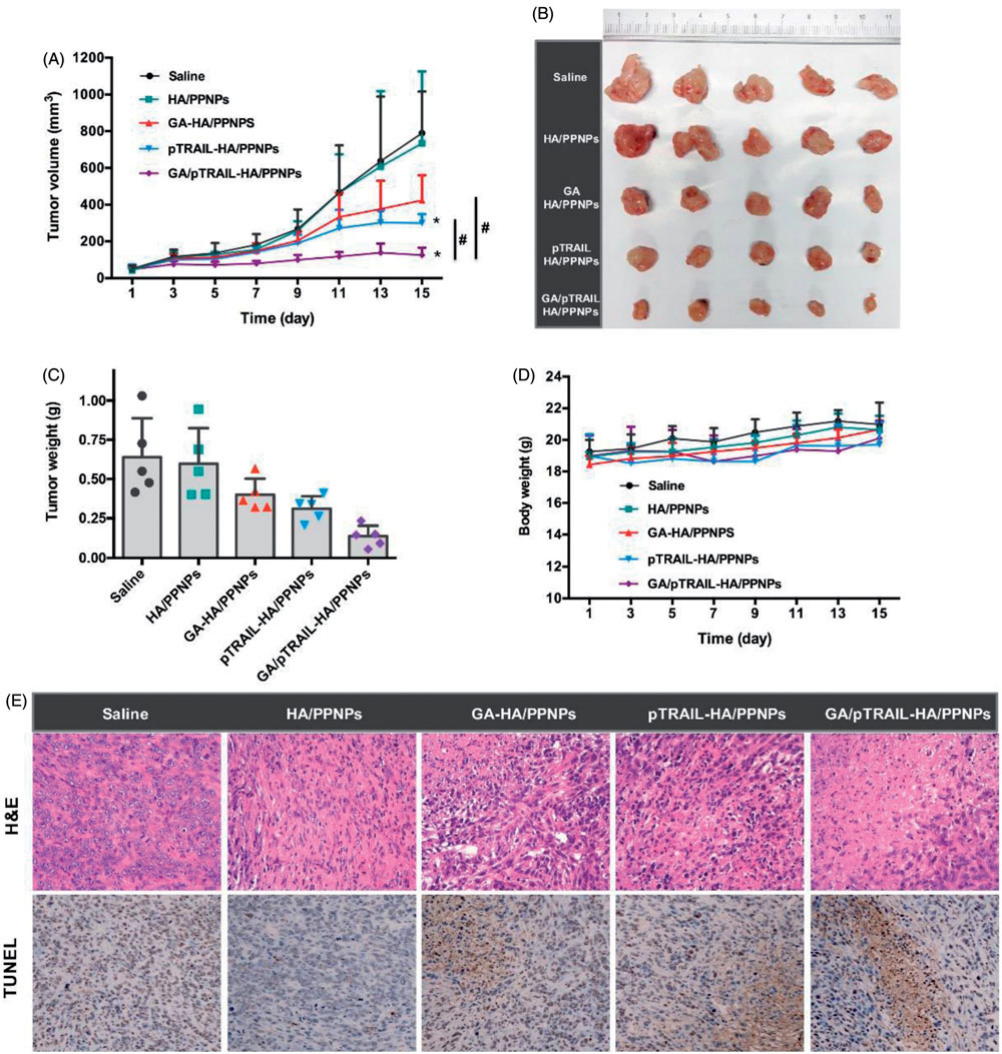
*In vivo* anti-tumor effect of GA/pTRAIL-HA/PPNPs. (A) Change of tumor volume in 4T1 cell-bearing mice after intravenous injection of different formulations. #*p <* .05 and *p < .05 vs. saline control. (B) Images of excised tumors of all groups at the end of study. (C) Change of body weight in 4T1-bearing mice during the study. (D) Tumor weight measured at the end of study (15 days post the initiation of treatment). Points are presented as mean ± SEM (*n* = 5). (E) The excised tumors of all groups were fixed and subjected to H&E histological staining and TUNEL immunohistochemical staining. Magnification ×400.

As controls, the tumor volumes of saline and HA/PPNPs gradually increased to 789 and 733 mm^3^ at day 15, respectively, showing negligible tumor inhibition effect of HA/PPNPs (Figure 6(A)). Both GA-HA/PPNPs and pTRAIL-HA/PPNPs treatment significantly inhibited the growth of tumors, while GA and pTRAIL co-loaded HA/PPNPs exhibited the strongest effect in inhibiting tumor growth. The tumor suppression rate (TSR) of GA-HA/PPNPs, pTRAIL-HA/PPNPs and GA/pTRAIL-HA/PPNPs were 46.4%, 62.0% and 84.1%, respectively. Tumors were collected at the end of study and weighted (Figure 6(B,C)). All the groups except HA/PPNPs group showed significantly lower tumor weight than that of saline control group, in which GA/pTRAIL-HA/PPNPs-treated group showed the lowest tumor weight. Moreover, throughout the therapeutic course, there was no obvious body weight loss in all the groups (Figure 6(D)).

We also performed H&E histological staining of the excised tumors to observe histopathological changes. As shown in Figure 6(E), the 4T1 tumors of saline and empty HA/PPNPs-treated mice showed few cell deaths. GA-HA/PPNPs and pTRAIL-HA/PPNPs group included several small necrotic areas. In contrast, GA/pTRAIL-HA/PPNPs-treated group presented a wide range of necrotic cell death across tumor. Taking all these *in vivo* results into account, we conclude that the HA/PPNPs-mediated co-delivery of GA and pTRAIL may be a promising strategy for combinatorial TNBC therapy.

## Conclusions

In conclusion, the present work reported the successful application of HA-coated PPNPs as a nanocarrier system to simultaneously deliver a natural compound GA and therapeutic pTRAIL to improve curative effect of TNBC. The nanocarrier improved selective uptake of the two loaded drugs in TNBC cells and promoted apoptosis of TNBC cells *both in vitro* and *in vivo*, resulting in more efficient combinatorial antitumor effect. This multifunctional NP system efficiently co-delivered GA and pTRAIL thus representing a promising therapy to treat TNBC by manipulating different molecular targets. This study also brings forth a platform strategy for co-delivery of therapeutic DNA and natural compounds in combinatorial TNBC therapy.
